# Interaction of Person-Affect-Cognition-Execution–Based Digital Interventions for Sleep Procrastination in Chinese University Students: Pilot Randomized Study

**DOI:** 10.2196/93920

**Published:** 2026-07-28

**Authors:** Huihui Xu, Liao Zhang, Xinyi Lai, Chengjia Zhao, Xiaoyun Ma, Guohua Zhang

**Affiliations:** 1 Department of Clinical Psychology, Wenzhou Medical University Wenzhou Seventh People's Hospital, Wenzhou, Zhejiang, China; 2 Zhejiang Provincial Clinical Research Center for Mental Disorders, The Affiliated Kangning Hospital of Wenzhou Medical University, Higher Education Park, Chashan Sub-district, Ouhai District, Wenzhou, Zhejiang, 325000, China, 86 13957764528; 3 Daliang Shunfeng Junior Middle School, Foshan, Guangdong, China; 4 School of Education, Renmin University of China, Beijing, China

**Keywords:** sleep procrastination, digital interventions, I-PACE model, Interaction of Person-Affect-Cognition-Execution, self-monitoring, university students

## Abstract

**Background:**

Sleep procrastination is a prevalent and detrimental behavior among university students. While digital interventions offer scalable solutions, they often lack a robust theoretical foundation and fail to differentiate themselves from clinical insomnia treatments.

**Objective:**

This pilot study examined the feasibility and preliminary effects of a novel intervention grounded in the Interaction of Person-Affect-Cognition-Execution (I-PACE) model, comparing online delivery with traditional offline delivery and a control group in reducing sleep procrastination.

**Methods:**

Forty-six students with high sleep procrastination were allocated to online (n=16), offline (n=16), or control (n=14) groups. The 8-week intervention targeted I-PACE components. Subjective and objective (wearable) measures were collected at baseline, postintervention, and a 2-month follow-up.

**Results:**

For subjective sleep procrastination, all groups improved similarly, with no significant group × time interaction. For objective sleep procrastination, a significant group × time interaction emerged (Wald *χ*²_16_=28.79, *P*=.03), with the online group showing numerically earlier sleep onset in later intervention weeks. The online group’s mean approached zero at week 7 (−0.05 h) but did not differ significantly from zero (*P*=.96) and rebounded at week 8. No between-group comparisons survived Bonferroni correction at any single week.

**Conclusions:**

This pilot study provides preliminary evidence that an app-based, I-PACE-grounded intervention is associated with a differential objective sleep timing trajectory. The specific mechanisms remain to be tested. Subjective improvements were comparable across conditions. Adequately powered trials with formal mediation testing are needed.

## Introduction

### Sleep Health and Sleep Procrastination Among University Students

Sleep health, conceptualized as a multidimensional pattern of sleep-wakefulness that supports physical and mental well-being and operationalized through the regularity, satisfaction, alertness, timing, efficiency, and duration (RU-SATED) model’s dimensions of regularity [1], satisfaction, alertness, timing, efficiency, and duration, is increasingly compromised in modern society [2]. In China, where adults average only 7.06 to 7.18 hours of nightly sleep [3], college students exhibit even more pronounced sleep deficits due to academic pressures, irregular schedules, and excessive digital engagement. Inadequate sleep in this group has been robustly linked to cognitive decline, emotional dysregulation, increased depression risk, and reduced academic performance [4-10]. A key contributor to inadequate sleep is bedtime procrastination, broadly defined as voluntarily delaying going to bed without external barriers [11]. For the purposes of this study, we draw a finer distinction, using the term “sleep procrastination” to refer specifically to the delay of actual sleep onset, as opposed to merely delaying the act of getting into bed [12]. This distinction is critical, as individuals may go to bed on time but continue to engage in activities that delay sleep itself [11,13]. Sleep procrastination is particularly prevalent among university students [14]. According to the China Sleep Research Report 2022, only about 6.4% of students reported going to bed as planned, while a striking 54.3% admitted to varying degrees of sleep procrastination [15]. Alarmingly, the latest 2025 report reveals a worsening trend, showing an overall increase in sleep procrastination within the Chinese population, now exacerbated by internet-related delays [3]. This progression underscores the escalating urgency of addressing this issue.

### Digital Interventions

Digital interventions have revolutionized the delivery of evidence-based sleep treatments, primarily for clinical insomnia. Internet- and app-based cognitive behavioral therapy for insomnia (CBT-I) has emerged as a highly effective and guideline-recommended nonpharmacological approach, demonstrating efficacy comparable to face-to-face therapy [16,17]. These programs typically integrate core CBT-I modules—psychoeducation/sleep hygiene, stimulus control, sleep restriction, and cognitive restructuring—often supplemented by relaxation techniques, to address the multifaceted nature of sleep disturbance [18-20].

However, this proliferation of digital tools stands in stark contrast to the landscape for sleep procrastination. As a prevalent but distinct behavioral issue, sleep procrastination lacks direct, theory-driven digital interventions. This gap presents a significant opportunity. The very technological advancements that underpin digital CBT-I—smartphone ubiquity, sophisticated app design, and the integration of wearable devices for objective sleep monitoring (eg, via photoplethysmography and accelerometry)—provide an ideal foundation for developing novel interventions [21,22]. Wearables, by offering continuous, objective measurement of sleep-wake patterns, can overcome the self-report biases inherent in studying voluntary sleep procrastination and enable personalized, real-time feedback [23]. The convergence of interactive digital platforms and objective biosensing thus creates a unique paradigm to address the specific, habitual, and context-driven nature of sleep procrastination. Critically, digital delivery may offer features that are theoretically conducive to behavior change—such as asynchronous access and wearable-integrated feedback—but whether these features confer advantages over face-to-face delivery in the context of sleep procrastination remains an open empirical question [21,23]. This theoretical perspective on how digital tools might work informs our comparative design between online and offline formats.

### Theoretical Framework: The Interaction of Person-Affect-Cognition-Execution Model for Sleep Procrastination

Accumulating evidence suggests that sleep procrastination is qualitatively distinct from clinical insomnia. Standard CBT-I, while effective for ameliorating core insomnia symptoms, often yields only modest improvements in delayed bedtime and sleep-onset behavior. This indicates that a different theoretical framework is required to understand and address the specific motivational and self-regulatory failures that characterize sleep procrastination [24]. The Interaction of Person-Affect-Cognition-Execution (I-PACE) model conceptualizes procrastination as an interaction of affective dysregulation, distorted cognitions, and impaired execution. Nighttime smartphone use often acts as maladaptive emotion regulation, reinforced through fear of missing out (FOMO) and habitual conditioning [25]. These individuals’ delayed circadian rhythms—regulated by endogenous physiological mechanisms with genetic underpinnings—interact with characteristic emotional dysregulation and negative cognitive biases to create a self-perpetuating cycle of nocturnal smartphone overuse [26]. Specifically, evening types demonstrate heightened susceptibility to negative affect, attentional bias toward sleep-related threats, and maladaptive beliefs about sleep consequences [27,28], which, according to Harvey’s cognitive model and procrastination repair theory, promote smartphone use as maladaptive regulation while simultaneously exacerbating FOMO and sleep-related anxiety [29]. This mechanistic pathway is supported by intervention studies demonstrating that techniques targeting these cognitive-affective components—including mental contrasting to address irrational beliefs and implementation intentions to reduce nighttime smartphone use—can significantly improve sleep timing [30,31]. Our study consequently examines circadian rhythm as a phenotypic marker influencing sleep procrastination through sequential mediation by sleep-specific affect, cognitive functions, and FOMO, all operating via increased problematic smartphone use—a testable pathway that aligns with both I-PACE theoretical predictions and established intervention outcomes.

### Aims and Hypotheses

Despite growing recognition of sleep procrastination as a significant public health concern, current interventions remain limited by several critical gaps: (1) many efforts rely predominantly on generic sleep hygiene education, yielding inconsistent outcomes; (2) there is a lack of theoretically grounded interventions specifically designed to target the underlying affective, cognitive, and behavioral mechanisms of sleep procrastination; (3) objective measurement tools, such as wearable devices, are rarely integrated to validate self-reported behavioral change; and (4) most fundamentally, existing approaches often fail to adequately differentiate sleep procrastination from clinical insomnia, leading to interventions that may be misaligned with the core problem.

Addressing these limitations, this study, building on the I-PACE model’s explanatory framework for sleep procrastination, aimed to design, pilot, and preliminarily evaluate a novel intervention that specifically targets its proposed pathways. This intervention was designed to modify key I-PACE components: sleep rhythms; the cognitive-affective (ie, sleep affect, sleep-related cognitive functions, and FOMO); and behavioral (ie, problematic smartphone use) pathways underlying sleep procrastination. This pilot study aimed to design, implement, and preliminarily evaluate an I-PACE-informed intervention for sleep procrastination. We compared online (app-based) and offline (group-based) delivery formats against a waitlist control, using both subjective measures and objective data from wearable fitness bands. Given the pilot nature of the trial, the study focused on estimating effect sizes and identifying trends rather than on definitive hypothesis testing.

H1: Format efficacy comparison. The online, app-based delivery of the I-PACE intervention will lead to significantly greater reductions in both subjective and objective measures of sleep procrastination at posttest and 2-month follow-up, compared to the offline group and control group.
H2: Mechanism of action—autonomy and self-monitoring. The superior outcomes of the online format will be mediated by 2 key process variables: (1) higher perceived autonomy support and (2) more frequent and consistent self-monitoring of sleep behavior, as facilitated by the digital platform.
H3: Theoretical pathway validation. Reductions in sleep procrastination across both intervention groups will be sequentially mediated by improvements in the I-PACE-specified mechanisms: namely, reductions in negative sleep affect and maladaptive sleep cognitions, leading to decreased problematic smartphone use at sleep time.

## Methods

### Participants

Participants were enrolled in a comprehensive study focused on sleep procrastination. They had to meet the following criteria: (1) university students aged 18 to 22 years; (2) a high level of sleep procrastination was determined through questionnaire assessment in this survey; (3) without severe pre-existing sleep disorders; and (4) having a relatively stable academic schedule. The sample consisted of 46 participants (n=42, 91% female; mean age 18.72, SD 0.83 y). The sample size (N=46) was determined based on feasibility for a pilot study rather than a formal power calculation. Subgroup sizes (n=14‐16 per group) are appropriate for exploratory comparisons but warrant cautious interpretation of the results

### Ethical Considerations

The study was approved by the Ethics Committee (Institutional Review Board) of Wenzhou Medical University (reference number 2022‐028). Written informed consent was obtained from all participants prior to their enrollment in the study. The consent form clearly explained the study purpose, procedures, potential risks, and the voluntary nature of participation. Participants were informed that they could withdraw at any time without penalty. All data were anonymized and stored in a password-protected database accessible only to the research team. Participant identifiers were removed and replaced with unique codes. Personal information was kept strictly confidential and used solely for the purposes of this study. Participants did not receive financial compensation. However, all participants in the intervention groups received a small token of appreciation (blessing cards) at the conclusion of the study.

### Measures

#### Participants’ Basic Information

A self-designed questionnaire was used to collect demographic information, including grade, gender, only-child status, and place of origin.

#### Bedtime Procrastination Scale

The Bedtime Procrastination Scale, developed by Kroese et al [11] and adapted by Xu [12], was used to measure sleep procrastination among college students. The scale consists of 8 items rated on a 5-point Likert scale (1=“never” to 5=“always”). Higher scores indicate a greater tendency to delay bedtime. The scale has demonstrated good reliability and validity and is suitable for Chinese college students [12], with Cronbach α coefficients of 0.87 at pretest, 0.88 at posttest, and 0.94 at follow-up assessment.

#### Problematic Mobile Phone Use Index

The Problematic Mobile Phone Use Index, developed by Leung et al [32], was used to assess problematic phone use among college students. The scale includes 4 dimensions: loss of control, withdrawal, escape, and inefficiency, with a total of 17 items rated on a 5-point Likert scale (1=“never” to 5=“always”). Higher scores indicate more severe problematic phone use. Participants with more than 8 affirmative responses were classified as problematic phone users. Huang et al [33] confirmed the scale’s reliability and validity among Chinese college students, with Cronbach α coefficients of 0.90 at pretest, 0.90 at posttest, and 0.90 at follow-up assessment.

#### Sleep Affect Scale

The Sleep Affect Scale, developed by Xu [12] based on qualitative research, was used to measure college students’ emotional experiences related to going to bed on time. The scale consists of 5 items rated on a 5-point Likert scale (1=“never” to 5=“always”). Higher scores indicate more negative emotions toward going to bed on time. The scale has demonstrated good reliability and validity and is suitable for Chinese college students, with Cronbach α coefficients of 0.88 at pretest, 0.87 at posttest, and 0.86 at follow-up assessment.

#### Sleep Beliefs and Attitudes Questionnaire

The Sleep Beliefs and Attitudes Questionnaire, developed by Morin et al [34] and revised by Yu [35], was used to measure sleep-related cognitive functions, including individuals’ beliefs and attitudes toward sleep. The scale includes 4 dimensions: expectations about sleep, worries and helplessness about insomnia, awareness of the impact of insomnia, and beliefs about medication, with a total of 12 items rated on a 5-point Likert scale (1=“strongly disagree” to 5=“strongly agree”). Higher scores indicate more irrational beliefs and distorted cognitions about sleep. The questionnaire has demonstrated good reliability and validity and is suitable for Chinese college students, with Cronbach α coefficients of 0.81 at pretest, 0.85 at posttest, and 0.81 at follow-up assessment.

#### College Students’ Fear of Missing Out Scale

The College Students’ Fear of Missing Out Scale, developed by Przybylski et al [36] and revised by Li et al [37], was used to measure the level of anxiety caused by the FOMO on others’ experiences. The scale includes 2 dimensions: fear of missing information and fear of missing situations, with a total of 8 items rated on a 5-point Likert scale (1=“strongly disagree” to 5=“strongly agree”). Higher scores indicate a higher level of FOMO. The scale has demonstrated good reliability and validity among Chinese college students [37], with Cronbach α coefficients of 0.88 at pretest, 0.90 at posttest, and 0.91 at follow-up assessment.

#### Morningness-Eveningness Questionnaire

The Morningness-Eveningness Questionnaire, developed by Adan and Almirall [38], was used to measure college students’ sleep rhythms. The scale consists of 5 multiple-choice questions, with scores classifying sleep rhythms into 5 types: definitely morning type (22‐25 points), moderately morning type (18‐21 points), intermediate type (12‐17 points), moderately evening type (8‐11 points), and definitely evening type (4‐7 points). The Chinese version of the scale has demonstrated good reliability and validity among Chinese college students [39], with Cronbach α coefficients of 0.60 at pretest, 0.64 at posttest, and 0.53 at follow-up assessment. Guilford considered reliability coefficients above 0.70 as high and 0.35 to 0.70 as acceptable [40]. The lower reliability in the sleep rhythm dimension may be due to its limited number of items, warranting future item additions.

#### Huawei Fitness Band

Each participant was provided with a Huawei fitness band (HUAWEI Band 7; Huawei Technologies Co Ltd) to monitor total nighttime sleep duration, actual sleep time, and actual wake-up time.

#### Participant-Owned Smartphones

Participants were required to have a smartphone capable of tracking total screen time.

#### Group Activity Self-Evaluation Scale

A self-designed Group Activity Self-Evaluation Scale was used for members of the offline intervention group to evaluate their experiences in group activities. The questionnaire consists of 10 objective questions and 3 subjective questions. Each objective question is rated on a 5-point Likert scale (1=“strongly disagree” to 5=“strongly agree”), evaluating 3 areas: overall satisfaction with the content, group process and atmosphere, and perceived changes in sleep procrastination and phone use. Three open-ended questions collected qualitative feedback on favorite sessions and personal gains.

#### Procedures

In this study, 200 participants with high levels of sleep procrastination were screened from a longitudinal study on sleep among Chinese university students from November 2021 to June 2022. Participants with a Bedtime Procrastination Scale score above the 75th percentile were identified as having high levels of sleep procrastination. Potentially eligible participants were sent invitation text messages containing a link to an online questionnaire for those interested in joining the intervention. The questionnaire clarified the group’s objectives, main content, schedule, format, and so on. The recruitment questionnaire outlined key details of the study, including its objectives, content, schedule, and format. It also assessed participants’ ability to commit to the intervention, including mandatory attendance at in-person group sessions for the offline arm. After collecting the questionnaires, the responses were screened based on their scores. Forty-eight individuals who met all inclusion criteria and consented to the time commitment were selected. Participants with the same baseline level were randomly divided into an offline intervention group, an online intervention group, and a control group.

### Intervention Description

#### Preintervention Phase

According to feasibility considerations for a pilot study, a total of 69 questionnaires were collected, and 48 participants were selected and randomized into 3 groups (1:1:1 ratio) after ensuring no significant baseline differences. Participants were interviewed to clarify the group’s objectives, content, schedule, and format, and they signed a group commitment form. Fitness bands with unique numbers were distributed, and participants were added to WeChat groups using their numbers as names. Two participants in the control group withdrew due to scheduling conflicts, resulting in a final sample of 46 participants (16 in-person, 16 online, and 14 control).

The team leader designed a sleep procrastination intervention for college students based on the I-PACE model, including group counseling and an online push notification plan. A backup plan was prepared for pandemic-related disruptions. Materials included an app for data collection, in-person tools (eg, consent forms, speakers, and art supplies), and online resources (eg, push notifications and a check-in app). The team confirmed the location (Group Psychological Counseling Room) and schedule (every Friday 7-8:30 PM, November 11-December 31, 2022) with the counseling center.

#### Intervention Phase

Starting on October 27, 2022, participants in all groups wore fitness bands for a 1-week trial to test the functionality of the objective data collection mini-program and the check-in mini-program for the online intervention group. This ensured that participants were familiar with the process.

#### Offline Intervention Group

The intervention consists of 8 consecutive chapters that can be completed during 8 weeks and cover all cognitive behavioral therapy components via the I-PACE model. Group counseling sessions helped members recognize negative emotions and cognitions related to sleep procrastination through structured activities such as cognitive restructuring using thought records and guided mindfulness exercises. Participants quantified their phone usage and purposes and were assigned homework to consciously reduce presleep phone use by setting implementation intentions (eg, “If it is 11 PM, then I will put my phone on airplane mode”). Facilitators guided members to share changes in feelings and cognitions within the group, creating positive feedback.

#### Online Intervention Group

Over 8 weeks, 2‐3 weekly posts were shared on topics such as sleep rhythm, sleep affect, sleep cognition, FOMO, problematic phone use, and sleep procrastination. The chapters are multimedia based, with short text passages, audio and video content, vignettes, and questions (multiple choice or free text) to foster active engagement with the intervention content. For example, a post on sleep cognition might include a short animated video explaining common sleep myths, followed by an interactive quiz. Tips were provided to reduce the negative impact of these factors on sleep procrastination. A check-in system included objective and subjective questions on the topic, such as “Did you try the ‘tech-free hour’ before bed last night?” and “Rate your level of anxiety about missing out on social media from 1 to 5.” The 3 members with the lowest total check-in scores prepared a post on sleep procrastination or phone use during the final week of the intervention.

#### Control Group

Only weekly data collection was conducted, with no additional interventions.

#### Postintervention Phase

Postintervention subjective measures were administered via an online survey platform distributed to all participants. For the offline intervention group, the final session was dedicated to consolidating gains and processing the termination experience. Facilitators guided members in reflecting on insights from the previous 7 sessions, addressed parting emotions, and completed the “Group Counseling Feedback Form.” As a token of appreciation, members received small blessing cards.

A follow-up assessment was conducted 2 months after the intervention (February 10‐17, 2023). All participants from the offline, online, and control groups were invited to wear the fitness bands again to record objective sleep indicators nightly. The online survey for subjective measures was distributed on February 17, 2023.

### Statistical Analyses

Given the pilot and exploratory nature of this study, analyses focused on estimating effect sizes and identifying trends rather than definitive hypothesis testing. All analyses were performed using SPSS Statistics software (version 26; IBM Corp), with a 2-tailed α of .05 and Bonferroni correction applied to all post hoc pairwise comparisons.

Primary outcomes—subjective sleep procrastination and objective sleep procrastination—were analyzed using generalized estimating equations (GEE) with an unstructured working correlation matrix. For subjective sleep procrastination, the model included group, time (baseline, postintervention, and follow-up), and the group × time interaction. For objective sleep procrastination, the model included group, time (weeks 1‐8 and follow-up week), and the group × time interaction. When a significant omnibus interaction was detected, simple effects were examined via estimated marginal means with Bonferroni correction, restricted to within-group comparisons of each follow-up time point against baseline (week 1). Results are reported as unstandardized regression coefficients (B) with 95% Wald CIs.

Secondary outcomes (problematic phone use, sleep affect, sleep-related cognitive functions, FOMO, and sleep rhythms) were assessed at the same 3 time points. Given the pilot nature of the study and the risk of inflated type I error from multiple comparisons, no inferential models were fitted for these variables. Part of the descriptive statistics are presented in the main text (Table 1); full results are provided in Multimedia Appendix 1.

**Table 1.** Observed means (with SDs in text) and estimated marginal means (95% Wald CIs) for primary outcomes from generalized estimating equation models, assessed at pretest, posttest, and 2-month follow-up in a pilot study among Chinese university students (N=46).

Outcome and group	Pretest EMM^a^ (95% CI)	Posttest EMM (95% CI)	Follow-up test EMM (95% CI)
SSP^b^			
Offline	32.50 (30.46-34.54)	29.13 (28.02-30.23)	33.42 (31.93-34.92)
Online	33.38 (31.18-35.57)	27.19 (24.22-30.26)	30.75 (27.79-33.71)
Control	31.86 (29.87-33.85)	26.21 (24.23-33.85)	32.00 (29.61-34.39)
OSP^c^			
Offline	1.01 (0.74-1.28)	1.42 (0.76-2.08)	1.61 (1.10-2.12)
Online	0.96 (0.73-1.19)	0.71 (0.01-1.40)	1.43 (0.60-2.26)
Control	1.17 (0.82-1.52)	1.17 (0.56-1.78)	1.12 (0.68-1.56)

a EMM: estimated marginal mean.

b SSP: subjective sleep procrastination.

c OSP: objective sleep procrastination.

## Results

### Primary Outcomes: Sleep Procrastination

#### Subjective Sleep Procrastination

**Figure 1.**
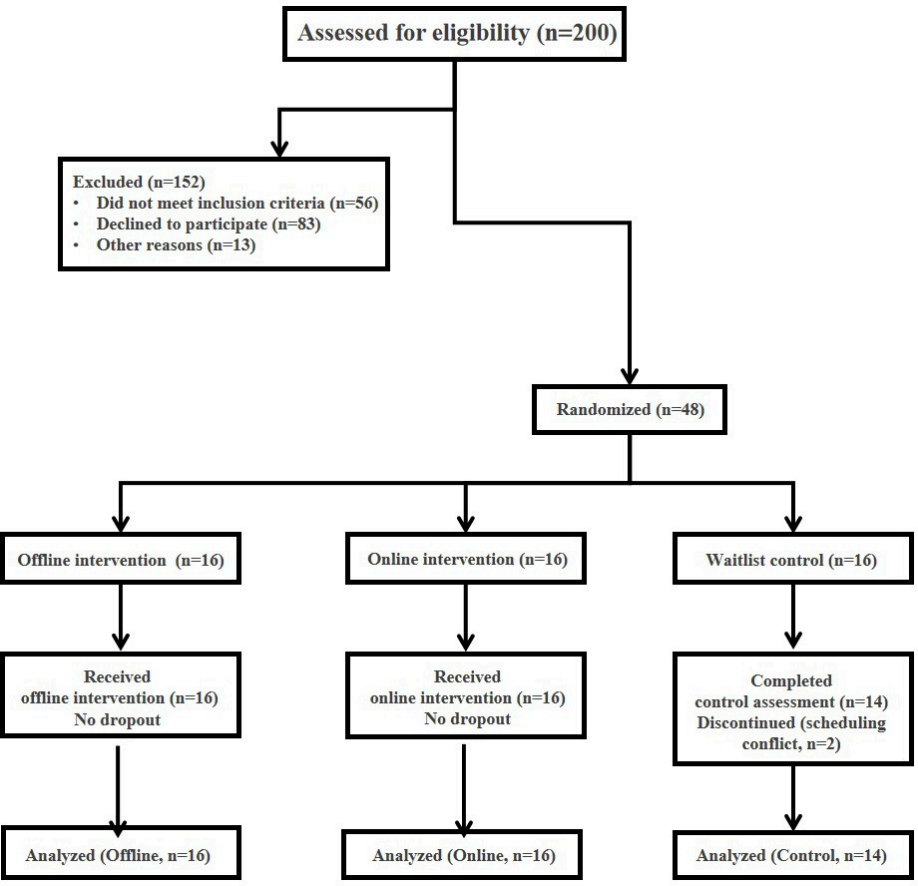
Flowchart of sample screening and intervention phase.

**Figure 2.**
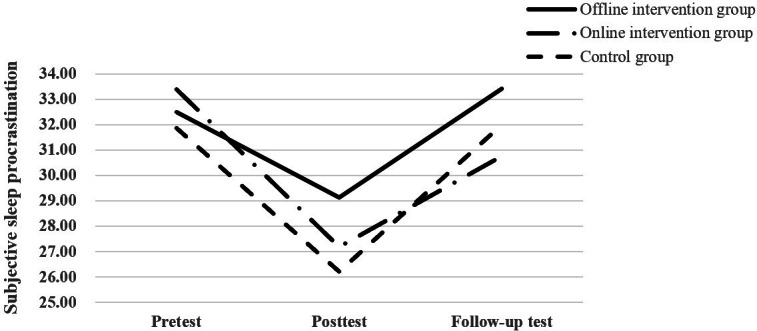
Subjective sleep procrastination scores at baseline, postintervention (week 8), and 2-month follow-up in a pilot randomized trial of an Interaction of Person-Affect-Cognition-Execution–based sleep procrastination intervention among Chinese university students (N=46).

Subjective sleep procrastination was assessed across the 3 groups at baseline, postintervention, and at the 2-month follow-up. All groups showed a reduction in scores immediately after the intervention. Details of the 3 groups are presented in Table 2. Figure 1 shows the flowchart of sample screening and the intervention phase. The online intervention group demonstrated the largest decrease, with scores falling from 33.38 (SD 4.63) to 27.19 (SD 6.48). The offline group decreased from 32.50 (SD 4.29) to 29.12 (SD 2.33), and the control group from 31.86 (SD 3.94) to 26.21 (SD 3.93). At the 2-month follow-up, scores rebounded across all groups, but the online group maintained the numerically lowest level (mean 30.75, SD 6.23), compared to the offline group (mean 33.40, SD 3.07) and the control group (mean 32.00, SD 4.74). This pattern suggests that the online intervention produced the most substantial initial improvement and the best-sustained effect (Figure 2).

A GEE model with an unstructured working correlation matrix was fitted to these data. The group ×time interaction was not significant (Wald *χ*²_4_=3.53, *P*=.47), indicating that the 3 groups did not show statistically distinguishable trajectories of change. Post hoc pairwise comparisons with Bonferroni correction further confirmed that no 2 groups differed significantly from each other at any single time point (all adjusted *P* values >.05; see Table 1 for estimated marginal means and Multimedia Appendix 1 for full pairwise contrasts). These results suggest that the subjective improvements observed were largely comparable across the 3 conditions and that the numerical advantage of the online group did not reach statistical significance.

**Table 2.** Demographic characteristics of participants at baseline in a pilot study of an Interaction of Person-Affect-Cognition-Execution–based model sleep procrastination intervention among Chinese university students (N=46).

Characteristic	Comparison of experimental and control groups		
	Offline intervention (n=16), n	Online intervention (n=16), n	Control group (n=14), n
Grade			
Freshman	14	5	6
Sophomore	2	11	8
Gender			
Male	1	2	1
Female	15	14	13
One-child family			
Yes	4	8	3
No	12	8	11
Residence			
Urban	6	8	4
Rural	10	8	10

#### Objective Sleep Time Procrastination

**Figure 3.**
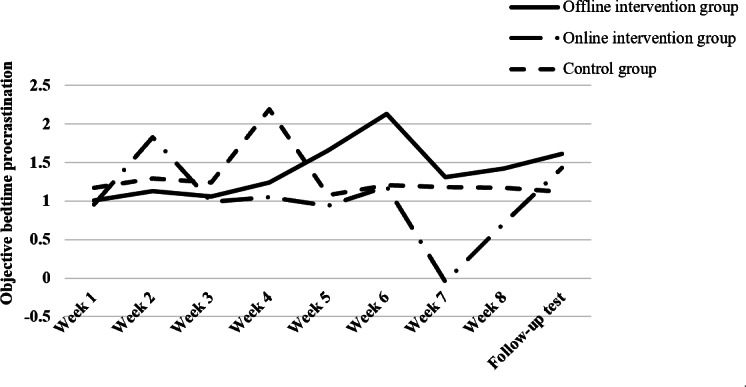
Weekly objective sleep procrastination across the 8-week intervention and a 2-month follow-up in a pilot study of an Interaction of Person-Affect-Cognition-Execution–based model sleep procrastination intervention among Chinese university students. Values above zero indicate delay relative to planned sleep time; values below zero indicate earlier-than-planned sleep onset (N=46).

Objective sleep procrastination was operationalized as the difference between actual and planned sleep times (ie, actual sleep time − planned sleep time). Weekly average values are plotted in Figure 3. The control group remained relatively stable at a positive delay, with positive delay values fluctuating around 1.0 to 1.2 hours. The offline intervention group showed fluctuations, peaking at a delay of 2.13 (SD 1.11) hours in week 6. The online intervention group, in contrast, exhibited marked improvement during the final weeks of the intervention. At week 7, the average score reached −0.05 (SD 3.24) hours, although this shift was not significantly different from zero (1-sample *t*_15_=−0.06, *P*=.96) and was not maintained at week 8, when the online group’s score returned to 0.71 (SD 1.47) hours. At the intervention end point (week 8), the online group’s objective procrastination remained numerically lower than that of the offline group (1.42 h, SD 1.38) and the control group (1.17 h, SD 1.21). During the follow-up period, the online group’s delay increased to 1.43 (SD 1.75) hours, still remaining numerically lower than the offline group’s 1.61 (SD 1.07) hours.

A GEE model with an unstructured working correlation matrix was applied across the 9 weekly data points. A significant group × time interaction was observed (Wald *χ²*_16_=28.79, *P*=.03), indicating that the temporal trajectories of objective sleep delay differed among the 3 groups. To decompose the significant group × time interaction, we conducted post hoc simple effect analyses restricted to within-group comparisons of each follow-up time point against baseline (week 1), using the same GEE model. For the offline group, objective sleep delay at week 6 was significantly higher than at baseline (95% CI −1.97 to −0.26, *P*=.001), consistent with the peak delay observed in Figure 3. No other within-group comparisons reached statistical significance in the offline group. For the online and control groups, none of the within-group comparisons against baseline survived Bonferroni correction (all adjusted *P* values >.05; see Multimedia Appendix 1 for full contrasts).

### Secondary Outcomes

Descriptive statistics for secondary outcomes—problematic phone use, sleep affect, sleep-related cognitive functions, FOMO, and sleep rhythms—at the 3 assessment points are presented in Table 1, with full details in Multimedia Appendix 1. Overall, the means of these variables followed a trajectory similar to that of subjective sleep procrastination: modest pre-to-post improvements were observed across all 3 groups, followed by a partial rebound at follow-up. The online group exhibited numerically more favorable postintervention scores on several measures (eg, FOMO and problematic phone use), though these differences were small in magnitude and, consistent with the primary outcome, not further examined via inferential models to avoid inflated type I error in a pilot sample and due to the lack of surviving pairwise comparisons. These descriptive patterns are broadly compatible with the I-PACE model’s prediction that changes in sleep-related affect, cognition, and smartphone behavior co-occur with reductions in sleep procrastination.

## Discussion

### Summary of Findings

This pilot randomized study provides initial evidence that a brief, I-PACE-based mobile program can reduce sleep procrastination in university students and that these gains persist for at least 2 months. The face-to-face version produced short-term benefits, but they faded after the group ended. Objective data corroborated self-reports and revealed a small subset of online participants who went to bed earlier than planned, suggesting goal surpassing rather than mere compliance. Importantly, this study was designed as a pilot trial to test feasibility and preliminary effects, not to formally test mediation. However, the intervention content was systematically derived from the I-PACE model, targeting its core components (sleep rhythms, affect, cognition, FOMO, and problematic phone use). The observed differential objective trajectory for the online group is consistent with the model’s predictions, providing a rationale for future adequately powered mediation studies. The following discussion therefore offers hypothesis-generating interpretations rather than evidence of confirmed mechanisms.

### Differential Trajectories in Subjective and Objective Sleep Procrastination

This pilot study compared an I-PACE-based online intervention, a content-matched offline group intervention, and a waitlist control on sleep procrastination in Chinese university students. The most notable finding was a significant group × time interaction for objectively measured sleep procrastination, indicating that the 3 groups’ trajectories of objectively recorded sleep delay differed over the course of the study. The online group showed a pattern of nonsignificant numerical improvement during the final intervention weeks, with objective delay decreasing across weeks 6‐8 and remaining numerically lower than the other 2 groups. However, this pattern requires cautious interpretation.

First, within-group comparisons revealed that only the offline group showed a significant change from baseline, while the online and control groups showed no significant within-group changes. The online group’s objective procrastination at week 7 briefly approached zero (−0.05 h), but a 1-sample *t* test confirmed that this value did not differ significantly from zero. At week 8, the online group’s value had returned to 0.71 hours, further indicating the transient nature of the week 7 shift. Second, the control group’s relative stability and the offline group’s fluctuating pattern—including a peak delay of 2.13 hours at week 6—suggest that the online format may have facilitated a steadier trajectory of improvement, but the small sample size and lack of surviving pairwise contrasts preclude strong claims about superiority. Notably, at the intervention end point (week 8), the 3 groups’ objective sleep procrastination values had largely converged, with all groups averaging approximately 1 hour of delay. For the unexpected increase in the offline group, this transient worsening coincided with a tightening of COVID-19 containment measures on campus, which suspended in-person classes and disrupted daily routines. The mandatory weekly group sessions may have also contributed to fatigue or perceived burden during that period. Importantly, this elevation returned to nonsignificant levels by week 8, suggesting a temporary perturbation rather than a sustained negative effect. Such external schedule instability may have independently shifted sleep timing across all groups, potentially masking any end point separation that the online intervention might otherwise have produced. The significant omnibus group × time interaction, observed despite this external disruption, therefore indicates that the online group’s trajectory differed primarily in its shape—showing a more pronounced late-intervention improvement before converging with the other groups under shared environmental constraints.

For subjective sleep procrastination, the group × time interaction was not significant, and no between-group comparison at any single time point survived Bonferroni correction. All 3 groups, including the waitlist control, reported substantial pre-to-post reductions, followed by a partial rebound at follow-up. This pattern is consistent with nonspecific effects—heightened self-monitoring from wearing a fitness band, repeated questionnaire completion, and Hawthorne effects—contributing to subjective improvement across conditions. The divergence between objective and subjective outcomes underscores the value of incorporating wearable-based measures when evaluating behavioral sleep interventions: objective indices may detect subtle treatment-related signals that self-report cannot reliably capture in a small pilot sample.

In sum, the online group’s objective trajectory was statistically distinguishable from the other groups’ trajectories in an omnibus sense, but the effect was not concentrated enough to survive conservative single-week comparisons. The findings provide preliminary evidence that app-based delivery may support a modest, distributed improvement in sleep timing while also demonstrating that much of the subjective benefit in such trials may be attributable to measurement reactivity.

### The Online Format Might Have Produced a Different Objective Trajectory—Hypotheses for Future Investigation

The online group’s more favorable objective trajectory during the final intervention weeks invites consideration of which design features may have contributed to this pattern.

A first hypothesis concerns autonomy support. One possibility is that the online intervention’s asynchronous and self-paced nature may have allowed participants to engage with materials at times that suited their schedules and energy levels. According to self-determination theory, such structural flexibility can satisfy the need for autonomy and thereby promote internalization of behavioral goals [41-43]. The offline group, by contrast, attended fixed weekly sessions—a format that, despite providing social support, may have been experienced as an external obligation by some participants. Future studies could directly test this hypothesis by administering validated autonomy-support scales (eg, the Basic Psychological Need Satisfaction Scale) at multiple time points and examining whether changes in perceived autonomy mediate the relationship between delivery format and sleep outcomes.

A second hypothesis involves self-monitoring and self-efficacy [21-23]. It is possible that the online platform’s integration with wearable-derived objective feedback might have provided participants with daily, personalized data on their sleep timing. From a social cognitive perspective [44], such concrete feedback can serve as a source of mastery experience—a primary contributor to self-efficacy beliefs. Witnessing objective evidence of reduced sleep delay may have reinforced participants’ confidence in their ability to regulate bedtime, creating a positive feedback loop. The offline group also wore fitness bands but did not receive the same level of automated, app-based feedback integration, which may have limited their opportunities to convert objective improvements into perceived self-efficacy gains.

A third set of factors relates to engagement. The online program used multimedia content, interactive quizzes, and a social accountability mechanism (the 3 lowest-scoring members prepared a post), which may have sustained engagement across the 8-week period. Research on digital behavior-change interventions suggests that such interactive features can increase adherence and habit formation. Whether these engagement features, autonomy support, or continuous feedback represent the “active ingredient”—or whether their combination is necessary—remains an open empirical question.

Critically, the current data do not allow us to differentiate among these hypotheses. The nonsignificant group × time interactions for secondary outcomes—problematic phone use, sleep affect, sleep beliefs, FOMO, and sleep rhythms (see the Secondary Outcomes subsection of the Results and Multimedia Appendix 1)—suggest that the intervention’s effects on the proposed I-PACE pathways were not strong enough to be detected with this sample. Future adequately powered trials should include repeated measures of these hypothesized mediators and employ formal tests of indirect effects (eg, multilevel structural equation modeling) to determine whether changes in autonomy, self-efficacy, or specific I-PACE components statistically account for the online format’s differential objective trajectory.

### Limitations and Future Directions

Several limitations must be considered when interpreting these findings. This was a pilot feasibility study with a modest sample size (N=46, n=14‐16 per group) not powered to detect small-to-moderate effects or to conduct robust subgroup analyses. The nonsignificant group × time interaction for subjective sleep procrastination and the absence of surviving corrected pairwise comparisons in objective outcomes should therefore be regarded as reflections of insufficient statistical power rather than definitive evidence of no effect. The estimated means and CIs reported in Table 1 provide effect-size benchmarks for sample-size planning in future trials.

The 2-month follow-up precludes assessment of long-term maintenance, particularly important given sleep procrastination’s chronic nature and the rebound observed in all groups at follow-up. Our sample, while adequately characterized for primary analyses, was insufficient for examining potential moderators such as gender, chronotype, or baseline severity factors. The absence of direct measures of autonomy support, self-efficacy, or intrinsic motivation means that the mechanistic hypotheses advanced in the “Why Might the Online Format Have Produced a Different Objective Trajectory? Hypotheses for Future Investigation” section remain speculative. The I-PACE model informed intervention content, but changes in its core components were assessed only descriptively, and no formal mediation analyses were performed. Future research should incorporate validated scales for autonomy and self-efficacy, implement repeated assessments of I-PACE variables across the intervention period, and test whether these variables statistically mediate the relationship between delivery format and outcomes.

Additionally, the study was conducted during the COVID-19 pandemic, a period when university students’ daily rhythms were inevitably disrupted. Postpandemic replication is warranted to assess generalizability. The study design cannot disentangle intervention effects from nonspecific monitoring effects because all groups wore fitness bands and completed repeated questionnaires. Future trials should include a no-monitoring control group (eg, no wearable, only pre-post assessments) to quantify the pure Hawthorne effect. Additionally, an active control group (eg, sleep hygiene education without I-PACE components) would help isolate the specific contribution of the I-PACE model. Finally, the demonstrated feasibility of the app-based platform suggests that future iterations could integrate just-in-time adaptive interventions using real-time wearable data, potentially enhancing personalization and scalability.

In summary, the present findings should be considered preliminary and hypothesis-generating. They provide initial evidence that a theory-grounded digital intervention can alter the trajectory of objectively measured sleep procrastination while also demonstrating that subjective self-report may be substantially influenced by nonspecific factors in such trials. Larger, adequately powered randomized controlled trials with formal mediation testing are needed to establish efficacy and to identify the active mechanisms of digital delivery.

### Conclusion

This pilot randomized study provides preliminary evidence that an I-PACE-informed app-based intervention is associated with a differential trajectory of objectively measured sleep procrastination in Chinese university students. The online group’s objective sleep delay decreased during the final intervention weeks, yielding a significant omnibus group × time interaction, although the effect was not strong enough to survive conservative single-week corrected comparisons. Subjective sleep procrastination improved across all groups, with no differential pattern detected, suggesting that nonspecific factors such as self-monitoring and measurement reactivity contributed substantially to perceived gains.

These findings underscore the value of incorporating objective outcome measures in behavioral sleep intervention research and highlight the need for caution when interpreting self-reported improvements in trials that involve wearable devices or frequent assessment. The hypotheses that autonomy-supportive design and continuous feedback may have contributed to the online format’s objective advantage remain speculative and require direct testing in future studies.

Three priorities emerge for subsequent research: (1) conducting fully powered trials with sample sizes informed by the effect estimates reported here; (2) incorporating validated measures of autonomy support, self-efficacy, and I-PACE components to test the hypothesized mechanisms through formal mediation analyses; and (3) determining which participant characteristics (eg, chronotype and baseline severity) predict differential response to digital vs face-to-face delivery. As sleep procrastination continues to affect a substantial proportion of university students, the development of scalable, evidence-based digital interventions remains an important public health goal, but definitive conclusions about their efficacy must await rigorous replication in larger samples.

## Supplementary material

10.2196/93920 Multimedia Appendix 1 Descriptive statistics (means and SDs) for secondary outcomes.

## Abbreviations

CBT-I: cognitive behavioral therapy for insomnia
FOMO: fear of missing out
GEE: generalized estimating equation
I-PACE: Interaction of Person-Affect-Cognition-Execution
RU-SATED: regularity, satisfaction, alertness, timing, efficiency, and duration
