# Supplementary material for: Interaction of Person-Affect-Cognition-Execution–Based Digital Interventions for Sleep Procrastination in Chinese University Students: Pilot Randomized Study
Source: JMIR Form Res. 2026 Jul 28;10:e93920. doi: 10.2196/93920 (PMC13412012; doi:10.2196/93920)
Supplement: Multimedia Appendix 1 [file formative-v10-e93920-s001.docx]

**Appendix**

**Table S1 Descriptive statistics (mean, standard deviation) and Kruskal-Wallis test results for subjective sleep procrastination, problematic phone use, sleep affect, sleep-related cognitive functions, fear of missing out, and sleep rhythms across three groups at pretest, post-test, and follow-up in a pilot randomized trial (N=46)**

|  | | Offline | | Online | | Control | |  | | |
| --- | --- | --- | --- | --- | --- | --- | --- | --- | --- | --- |
|  |  | *M* | *SD* | *M* | *SD* | *M* | *SD* | *H* | *p* |  |
| Pretest | SP | 32.50 | 4.29 | 33.38 | 4.63 | 31.86 | 3.94 | 1.10 | 0.578 |  |
|  | PPU | 62.00 | 10.13 | 60.25 | 13.25 | 60.14 | 11.93 | 0.23 | 0.892 |  |
|  | SA | 13.50 | 4.91 | 16.00 | 4.26 | 15.86 | 4.28 | 2.37 | 0.306 |  |
|  | SBA | 37.69 | 4.57 | 38.75 | 7.96 | 34.93 | 5.34 | 2.70 | 0.259 |  |
|  | FoMo | 26.56 | 6.98 | 27.50 | 8.56 | 28.64 | 5.98 | 0.49 | 0.784 |  |
|  | SR | 14.13 | 2.53 | 13.75 | 3.24 | 12.79 | 2.22 | 3.72 | 0.156 |  |
| Post-test | SP | 29.13 | 2.33 | 27.19 | 6.48 | 26.21 | 3.93 | 6.13 | 0.047 | a>b,c |
|  | PPU | 63.50 | 5.30 | 60.06 | 10.11 | 58.93 | 9.29 | 3.28 | 0.194 |  |
|  | SA | 15.88 | 2.73 | 15.69 | 3.77 | 16.43 | 2.56 | 2.14 | 0.343 |  |
|  | SBA | 36.25 | 6.09 | 37.06 | 7.38 | 35.93 | 6.04 | 0.24 | 0.888 |  |
|  | FoMo | 25.13 | 4.91 | 26.88 | 7.25 | 28.21 | 6.39 | 1.70 | 0.428 |  |
|  | SR | 15.50 | 1.51 | 14.06 | 1.44 | 15.07 | 2.06 | 7.07 | 0.029 | b<a |
| Follow-up test | SP | 33.40 | 3.07 | 30.75 | 6.23 | 32.00 | 4.74 | 1.37 | 0.503 |  |
|  | PPU | 62.93 | 6.51 | 54.50 | 18.62 | 59.14 | 13.05 | 2.02 | 0.364 |  |
|  | SA | 16.07 | 4.25 | 15.75 | 5.40 | 17.79 | 4.48 | 1.47 | 0.480 |  |
|  | SBA | 36.07 | 5.39 | 35.13 | 7.25 | 37.00 | 7.36 | 0.30 | 0.862 |  |
|  | FoMo | 25.93 | 6.15 | 25.25 | 8.77 | 28.36 | 6.88 | 0.83 | 0.660 |  |
|  | SR | 13.33 | 2.82 | 14.13 | 2.78 | 12.79 | 2.81 | 2.27 | 0.322 |  |

Note: SP = Sleep Procrastination, PPU = Problematic Phone Use, SA = Sleep Affect, SBA = Sleep Beliefs and Attitudes, FoMo = Fear of Missing Out, SR = Sleep Rhythms. The same below.

**Table S2 Descriptive statistics (mean, standard deviation) and Friedman test results for subjective measures (sleep procrastination, problematic phone use, sleep affect, sleep-related cognitive functions, fear of missing out, sleep rhythms) across three groups at pretest, post-test, and follow-up in a pilot study (N=46)**

|  | Offline | | | Online | | | Control | | |
| --- | --- | --- | --- | --- | --- | --- | --- | --- | --- |
|  | *χ*² | *p* |  | *χ*² | *p* |  | *χ*² | *p* |  |
| SP | 10.29 | **0.006** | 2<1,3 | 8.49 | **0.014** | 2<1 | 11.39 | **0.003** | 2<1,3 |
| PPU | 1.73 | 0.420 |  | 1.41 | 0.494 |  | 1.00 | 0.607 |  |
| SA | 2.67 | 0.264 |  | 0.10 | 0.953 |  | 2.47 | 0.291 |  |
| SBA | 3.21 | 0.201 |  | 0.53 | 0.769 |  | 0.51 | 0.775 |  |
| FoMo | 1.66 | 0.436 |  | 0.13 | 0.937 |  | 0.79 | 0.673 |  |
| SR | 9.77 | **0.008** | 3<2 | 0.29 | 0.865 |  | 8.64 | **0.013** | 1,3<2 |

Note: 1 = Pretest, 2 = Post-test, 3 = follow-up test.

**Table S3 Descriptive statistics (mean, standard deviation) and Kruskal-Wallis test results for objective sleep measures, including planned sleep time, actual sleep time, objective sleep procrastination, and average daily phone use duration, across three groups during the 8-week intervention and follow-up week in a pilot study (N=46)**

|  | | Offline | | Online | | Control | |  | | |
| --- | --- | --- | --- | --- | --- | --- | --- | --- | --- | --- |
|  |  | *M* | *SD* | *M* | *SD* | *M* | *SD* | *H* | *p* |  |
| Week 1 | PB | -0.06 | 0.51 | -0.27 | 0.90 | -0.39 | 0.49 | 2.71 | 0.258 |  |
|  | AB | 0.95 | 0.87 | 0.69 | 1.11 | 0.78 | 0.71 | 0.88 | 0.644 |  |
|  | OSP | -1.01 | 0.57 | -0.96 | 0.49 | -1.17 | 0.70 | 0.65 | 0.723 |  |
|  | ADPUD | 11.99 | 12.02 | 9.57 | 2.33 | 9.69 | 2.04 | 0.18 | 0.913 |  |
| Week 2 | PB | 0.09 | 0.84 | -0.90 | 3.19 | -0.17 | 0.72 | 1.07 | 0.586 |  |
|  | AB | 1.22 | 0.93 | 0.94 | 1.22 | 1.11 | 1.01 | 1.06 | 0.589 |  |
|  | OSP | -1.13 | 0.97 | -1.83 | 2.76 | -1.29 | 0.77 | 0.30 | 0.859 |  |
|  | ADPUD | 8.93 | 2.72 | 8.24 | 2.51 | 8.53 | 2.62 | 0.42 | 0.811 |  |
| Week 3 | PB | 0.09 | 0.61 | -0.21 | 0.83 | -0.27 | 0.42 | 2.92 | 0.233 |  |
|  | AB | 1.16 | 0.80 | 0.78 | 0.90 | 0.96 | 0.58 | 0.92 | 0.630 |  |
|  | OSP | -1.06 | 0.78 | -0.99 | 0.39 | -1.24 | 0.74 | 1.23 | 0.541 |  |
|  | ADPUD | 10.14 | 3.87 | 8.44 | 2.61 | 9.74 | 2.18 | 3.41 | 0.181 |  |
| Week 4 | PB | 0.07 | 0.56 | -0.24 | 0.87 | -0.97 | 3.52 | 1.79 | 0.409 |  |
|  | AB | 1.31 | 1.02 | 0.82 | 1.15 | 1.23 | 1.03 | 2.58 | 0.275 |  |
|  | OSP | -1.24 | 0.95 | -1.05 | 0.59 | -2.19 | 3.58 | 0.03 | 0.986 |  |
|  | ADPUD | 8.40 | 2.75 | 8.93 | 2.51 | 9.14 | 2.54 | 0.40 | 0.818 |  |
| Week 5 | PB | 0.27 | 0.70 | 0.08 | 0.93 | 0.07 | 0.70 | 1.39 | 0.498 |  |
|  | AB | 1.93 | 1.36 | 1.02 | 1.29 | 1.15 | 0.91 | 4.97 | 0.083 |  |
|  | OSP | -1.66 | 1.02 | -0.94 | 0.59 | -1.08 | 0.66 | 5.75 | 0.057 |  |
|  | ADPUD | 9.97 | 2.97 | 8.94 | 2.81 | 9.06 | 3.06 | 1.56 | 0.460 |  |
| Week 6 | PB | -0.13 | 0.87 | -0.16 | 0.85 | -0.06 | 0.66 | 0.65 | 0.723 |  |
|  | AB | 2.01 | 1.43 | 1.04 | 1.23 | 1.15 | 0.86 | 4.38 | 0.112 |  |
|  | OSP | -2.13 | 1.11 | -1.19 | 0.75 | -1.21 | 0.74 | 7.74 | **0.021** | a<b |
|  | ADPUD | 9.60 | 2.09 | 8.63 | 2.42 | 8.33 | 3.04 | 2.97 | 0.227 |  |
| Week 7 | PB | 0.41 | 2.66 | -0.51 | 0.97 | -0.58 | 1.25 | 2.22 | 0.330 |  |
|  | AB | 1.71 | 1.56 | -0.56 | 3.53 | 0.60 | 1.14 | 10.02 | **0.007** | b<a |
|  | OSP | -1.31 | 3.30 | 0.05 | 3.24 | -1.18 | 0.63 | 8.34 | **0.015** | a<b |
|  | ADPUD | 8.92 | 2.74 | 8.47 | 3.07 | 8.82 | 3.23 | 0.42 | 0.809 |  |
| Week 8 | PB | -0.28 | 0.91 | -0.79 | 0.87 | -0.46 | 1.01 | 2.03 | 0.363 |  |
|  | AB | 1.14 | 1.83 | -0.08 | 1.86 | 0.71 | 1.49 | 6.43 | **0.040** | b<a |
|  | OSP | -1.42 | 1.38 | -0.71 | 1.47 | -1.17 | 1.21 | 3.30 | 0.192 |  |
|  | ADPUD | 9.93 | 3.31 | 8.00 | 2.83 | 8.95 | 2.83 | 3.14 | 0.208 |  |
| Follow-up Week | PB | -0.03 | 0.64 | -0.59 | 1.21 | 0.18 | 0.72 | 4.49 | 0.106 |  |
|  | AB | 1.58 | 1.52 | 0.84 | 2.29 | 1.30 | 1.06 | 7.41 | **0.025** | b<a,c |
|  | OSP | -1.61 | 1.07 | -1.43 | 1.75 | -1.12 | 0.87 | 1.28 | 0.528 |  |
|  | ADPUD | 8.89 | 2.11 | 8.07 | 2.73 | 9.10 | 3.71 | 0.22 | 0.895 |  |

Note: PB = Planned Sleeptime, AB = Actual Sleeptime, OSP = Objective Sleep Procrastination, ADPUD = Average Daily Mobile Phone Usage Duration. The same below.

**Table S4 Descriptive statistics (mean, standard deviation) and Friedman test results for objective sleep measures (planned sleep time, actual sleep time, objective sleep procrastination, average daily phone use duration) across three groups during the 8-week intervention and follow-up week in a pilot study (N=46)**

|  | Offline | | | Online | | | Control | | |
| --- | --- | --- | --- | --- | --- | --- | --- | --- | --- |
|  | *χ*² | *p* |  | *χ*² | *p* |  | *χ*² | *p* |  |
| PB | 11.74 | 0.163 |  | 15.13 | 0.057 |  | 11.14 | 0.194 |  |
| AB | 16.09 | **0.041** | 1<5,6,7,9  2,8<6  5<8 | 25.68 | **0.001** | 8<2,3,4,5,6,9  7<2,4,5,6,9 | 8.99 | 0.343 |  |
| OSP | 16.25 | **0.039** | 1,2,3,4,8<6  1,2<7 | 15.41 | 0.052 |  | 3.66 | 0.818 |  |
| ADPUD | 10.61 | 0.225 |  | 12.39 | 0.134 |  | 13.04 | 0.111 |  |

Note: For pairwise comparisons, 1 = week 1, 2 = week 2, ..., and 9 = follow-up.

**Table S5 Full pairwise comparisons (Bonferroni corrected) for subjective sleep procrastination from a generalized estimating equation model in a pilot Study of an I-PACE-based sleep procrastination intervention among Chinese university students (N=46).**

| TIME*GROUP | | Mean Difference | SE | *df* | *p* | 95% Wald CI for Difference | |
| --- | --- | --- | --- | --- | --- | --- | --- |
|  |  |  |  |  |  | Lower | Upper |
| [TIME1]*[a] | [TIME1]*[b] | -0.88 | 1.53 | 1.00 | 0.57 | -3.87 | 2.12 |
|  | [TIME1]*[c] | 0.64 | 1.45 | 1.00 | 0.66 | -2.20 | 3.49 |
|  | [TIME2]*[a] | 3.38 | 1.21 | 1.00 | 0.01 | 1.01 | 5.74 |
|  | [TIME2]*[b] | 5.31 | 1.88 | 1.00 | 0.00 | 1.62 | 9.00 |
|  | [TIME2]*[c] | 6.29 | 1.45 | 1.00 | 0.00 | 3.45 | 9.13 |
|  | [TIME3]*[a] | -0.99 | 1.36 | 1.00 | 0.47 | -3.64 | 1.67 |
|  | [TIME3]*[b] | 1.75 | 1.83 | 1.00 | 0.34 | -1.84 | 5.34 |
|  | [TIME3]*[c] | 0.50 | 1.60 | 1.00 | 0.76 | -2.64 | 3.64 |
| [TIME1]*[b] | [TIME1]*[a] | 0.88 | 1.53 | 1.00 | 0.57 | -2.12 | 3.87 |
|  | [TIME1]*[c] | 1.52 | 1.51 | 1.00 | 0.32 | -1.45 | 4.48 |
|  | [TIME2]*[a] | 4.25 | 1.26 | 1.00 | 0.00 | 1.79 | 6.71 |
|  | [TIME2]*[b] | 6.19 | 1.30 | 1.00 | 0.00 | 3.64 | 8.73 |
|  | [TIME2]*[c] | 7.16 | 1.51 | 1.00 | 0.00 | 4.20 | 10.12 |
|  | [TIME3]*[a] | -0.11 | 1.38 | 1.00 | 0.94 | -2.82 | 2.60 |
|  | [TIME3]*[b] | 2.63 | 2.11 | 1.00 | 0.21 | -1.51 | 6.76 |
|  | [TIME3]*[c] | 1.38 | 1.66 | 1.00 | 0.41 | -1.87 | 4.62 |
| [TIME1]*[c] | [TIME1]*[a] | -0.64 | 1.45 | 1.00 | 0.66 | -3.49 | 2.20 |
|  | [TIME1]*[b] | -1.52 | 1.51 | 1.00 | 0.32 | -4.48 | 1.45 |
|  | [TIME2]*[a] | 2.73 | 1.16 | 1.00 | 0.02 | 0.46 | 5.01 |
|  | [TIME2]*[b] | 4.67 | 1.87 | 1.00 | 0.01 | 1.01 | 8.33 |
|  | [TIME2]*[c] | 5.64 | 1.53 | 1.00 | 0.00 | 2.64 | 8.65 |
|  | [TIME3]*[a] | -1.63 | 1.30 | 1.00 | 0.21 | -4.17 | 0.91 |
|  | [TIME3]*[b] | 1.11 | 1.82 | 1.00 | 0.54 | -2.46 | 4.67 |
|  | [TIME3]*[c] | -0.14 | 1.34 | 1.00 | 0.91 | -2.76 | 2.48 |
| [TIME2]*[a] | [TIME1]*[a] | -3.38 | 1.21 | 1.00 | 0.01 | -5.74 | -1.01 |
|  | [TIME1]*[b] | -4.25 | 1.26 | 1.00 | 0.00 | -6.71 | -1.79 |
|  | [TIME1]*[c] | -2.73 | 1.16 | 1.00 | 0.02 | -5.01 | -0.46 |
|  | [TIME2]*[b] | 1.94 | 1.67 | 1.00 | 0.25 | -1.33 | 5.21 |
|  | [TIME2]*[c] | 2.91 | 1.16 | 1.00 | 0.01 | 0.64 | 5.18 |
|  | [TIME3]*[a] | -4.36 | 0.89 | 1.00 | 0.00 | -6.11 | -2.61 |
|  | [TIME3]*[b] | -1.63 | 1.61 | 1.00 | 0.31 | -4.78 | 1.53 |
|  | [TIME3]*[c] | -2.88 | 1.35 | 1.00 | 0.03 | -5.51 | -0.24 |
| [TIME2]*[b] | [TIME1]*[a] | -5.31 | 1.88 | 1.00 | 0.00 | -9.00 | -1.62 |
|  | [TIME1]*[b] | -6.19 | 1.30 | 1.00 | 0.00 | -8.73 | -3.64 |
|  | [TIME1]*[c] | -4.67 | 1.87 | 1.00 | 0.01 | -8.33 | -1.01 |
|  | [TIME2]*[a] | -1.94 | 1.67 | 1.00 | 0.25 | -5.21 | 1.33 |
|  | [TIME2]*[c] | 0.97 | 1.87 | 1.00 | 0.60 | -2.69 | 4.63 |
|  | [TIME3]*[a] | -6.30 | 1.77 | 1.00 | 0.00 | -9.76 | -2.84 |
|  | [TIME3]*[b] | -3.56 | 1.89 | 1.00 | 0.06 | -7.27 | 0.14 |
|  | [TIME3]*[c] | -4.81 | 1.99 | 1.00 | 0.02 | -8.71 | -0.92 |
| [TIME2]*[c] | [TIME1]*[a] | -6.2857 | 1.45 | 1.00 | 0.00 | -9.13 | -3.45 |
|  | [TIME1]*[b] | -7.1607 | 1.51 | 1.00 | 0.00 | -10.12 | -4.20 |
|  | [TIME1]*[c] | -5.6429 | 1.53 | 1.00 | 0.00 | -8.65 | -2.64 |
|  | [TIME2]*[a] | -2.9107 | 1.16 | 1.00 | 0.01 | -5.18 | -0.64 |
|  | [TIME2]*[b] | -0.97 | 1.87 | 1.00 | 0.60 | -4.63 | 2.69 |
|  | [TIME3]*[a] | -7.2733 | 1.30 | 1.00 | 0.00 | -9.81 | -4.73 |
|  | [TIME3]*[b] | -4.5357 | 1.82 | 1.00 | 0.01 | -8.10 | -0.98 |
|  | [TIME3]*[c] | -5.7857 | 1.78 | 1.00 | 0.00 | -9.27 | -2.30 |
| [TIME3]*[a] | [TIME1]*[a] | 0.99 | 1.36 | 1.00 | 0.47 | -1.67 | 3.64 |
|  | [TIME1]*[b] | 0.11 | 1.38 | 1.00 | 0.94 | -2.60 | 2.82 |
|  | [TIME1]*[c] | 1.63 | 1.30 | 1.00 | 0.21 | -0.91 | 4.17 |
|  | [TIME2]*[a] | 4.3626 | 0.89 | 1.00 | 0.00 | 2.61 | 6.11 |
|  | [TIME2]*[b] | 6.3001 | 1.77 | 1.00 | 0.00 | 2.84 | 9.76 |
|  | [TIME2]*[c] | 7.2733 | 1.30 | 1.00 | 0.00 | 4.73 | 9.81 |
|  | [TIME3]*[b] | 2.74 | 1.71 | 1.00 | 0.11 | -0.62 | 6.09 |
|  | [TIME3]*[c] | 1.49 | 1.46 | 1.00 | 0.31 | -1.38 | 4.36 |
| [TIME3]*[b] | [TIME1]*[a] | -1.75 | 1.83 | 1.00 | 0.34 | -5.34 | 1.84 |
|  | [TIME1]*[b] | -2.63 | 2.11 | 1.00 | 0.21 | -6.76 | 1.51 |
|  | [TIME1]*[c] | -1.11 | 1.82 | 1.00 | 0.54 | -4.67 | 2.46 |
|  | [TIME2]*[a] | 1.63 | 1.61 | 1.00 | 0.31 | -1.53 | 4.78 |
|  | [TIME2]*[b] | 3.56 | 1.89 | 1.00 | 0.06 | -0.14 | 7.27 |
|  | [TIME2]*[c] | 4.5357 | 1.82 | 1.00 | 0.01 | 0.98 | 8.10 |
|  | [TIME3]*[a] | -2.74 | 1.71 | 1.00 | 0.11 | -6.09 | 0.62 |
|  | [TIME3]*[c] | -1.25 | 1.94 | 1.00 | 0.52 | -5.05 | 2.55 |
| [TIME3]*[c] | [TIME1]*[a] | -0.50 | 1.60 | 1.00 | 0.76 | -3.64 | 2.64 |
|  | [TIME1]*[b] | -1.38 | 1.66 | 1.00 | 0.41 | -4.62 | 1.87 |
|  | [TIME1]*[c] | 0.14 | 1.34 | 1.00 | 0.91 | -2.48 | 2.76 |
|  | [TIME2]*[a] | 2.8750 | 1.35 | 1.00 | 0.03 | 0.24 | 5.51 |
|  | [TIME2]*[b] | 4.8125 | 1.99 | 1.00 | 0.02 | 0.92 | 8.71 |
|  | [TIME2]*[c] | 5.7857 | 1.78 | 1.00 | 0.00 | 2.30 | 9.27 |
|  | [TIME3]*[a] | -1.49 | 1.46 | 1.00 | 0.31 | -4.36 | 1.38 |
|  | [TIME3]*[b] | 1.25 | 1.94 | 1.00 | 0.52 | -2.55 | 5.05 |

Note: TIME1 = Pretest, TIME2 = Post-test, TIME3 = Follow-up test., a = Offline Intervention Group, b = Online Intervention Group, c = Control Group.

**Table S6 Within‑group comparisons (Bonferroni corrected) for objective sleep procrastination from a generalized estimating equation model in a pilot study of an I-PACE-based sleep procrastination intervention among Chinese university students (N=46).**

|  | | Mean Difference | SE | *df* | *p* | 95% Wald CI for Difference | |
| --- | --- | --- | --- | --- | --- | --- | --- |
|  |  |  |  |  |  | Lower | Upper |
| Offline | [TIME1]*[TIME2] | -0.12 | 0.23 | 1.00 | 1.00 | -0.86 | 0.63 |
|  | [TIME1]*[TIME3] | -0.05 | 0.19 | 1.00 | 1.00 | -0.64 | 0.55 |
|  | [TIME1]*[TIME4] | -0.23 | 0.26 | 1.00 | 1.00 | -1.06 | 0.60 |
|  | [TIME1]*[TIME5] | -0.65 | 0.24 | 1.00 | 0.21 | -1.40 | 0.10 |
|  | [TIME1]*[TIME6] | -1.12 | 0.27 | 1.00 | 0.00 | -1.97 | -0.26 |
|  | [TIME1]*[TIME7] | -0.29 | 0.78 | 1.00 | 1.00 | -2.79 | 2.20 |
|  | [TIME1]*[TIME8] | -0.41 | 0.26 | 1.00 | 1.00 | -1.25 | 0.44 |
|  | [TIME1]*[TIME9] | -0.60 | 0.25 | 1.00 | 0.66 | -1.40 | 0.21 |
| Online | [TIME1]*[TIME2] | -0.87 | 0.66 | 1.00 | 1.00 | -2.99 | 1.26 |
|  | [TIME1]*[TIME3] | -0.03 | 0.10 | 1.00 | 1.00 | -0.34 | 0.29 |
|  | [TIME1]*[TIME4] | -0.09 | 0.12 | 1.00 | 1.00 | -0.46 | 0.28 |
|  | [TIME1]*[TIME5] | 0.03 | 0.15 | 1.00 | 1.00 | -0.45 | 0.50 |
|  | [TIME1]*[TIME6] | -0.23 | 0.17 | 1.00 | 1.00 | -0.78 | 0.32 |
|  | [TIME1]*[TIME7] | 1.01 | 0.76 | 1.00 | 1.00 | -1.42 | 3.44 |
|  | [TIME1]*[TIME8] | 0.26 | 0.33 | 1.00 | 1.00 | -0.79 | 1.30 |
|  | [TIME1]*[TIME9] | -0.47 | 0.42 | 1.00 | 1.00 | -1.81 | 0.87 |
| Control | [TIME1]*[TIME2] | -0.11 | 0.17 | 1.00 | 1.00 | -0.64 | 0.42 |
|  | [TIME1]*[TIME3] | -0.06 | 0.20 | 1.00 | 1.00 | -0.71 | 0.58 |
|  | [TIME1]*[TIME4] | -1.02 | 0.93 | 1.00 | 1.00 | -3.98 | 1.94 |
|  | [TIME1]*[TIME5] | 0.09 | 0.21 | 1.00 | 1.00 | -0.59 | 0.77 |
|  | [TIME1]*[TIME6] | -0.03 | 0.24 | 1.00 | 1.00 | -0.82 | 0.75 |
|  | [TIME1]*[TIME7] | 0.00 | 0.21 | 1.00 | 1.00 | -0.68 | 0.67 |
|  | [TIME1]*[TIME8] | 0.00 | 0.31 | 1.00 | 1.00 | -0.99 | 0.99 |
|  | [TIME1]*[TIME9] | 0.05 | 0.25 | 1.00 | 1.00 | -0.75 | 0.86 |

Note: TIME1 = week 1, TIME2 = week 2, ..., and TIME9 = follow-up.
